# Cytotoxin-Associated Gene A-Positive *Helicobacter pylori* Promotes Autophagy in Colon Cancer Cells by Inhibiting miR-125b-5p

**DOI:** 10.1155/2021/6622092

**Published:** 2021-03-02

**Authors:** Xiaolin Zhong, Ou Chen, TieJun Zhou, Muhan Lü, Juyi Wan

**Affiliations:** ^1^Department of Gastroenterology, The Affiliated Hospital of Southwest Medical University, Luzhou 646000, China; ^2^Nuclear Medicine and Molecular Imaging Key Laboratory of Sichuan Province, Luzhou 646000, China; ^3^Department of Gastroenterology, Ya'an People's Hospital, Ya'an 625000, China; ^4^Department of Pathology, The Affiliated Hospital of Southwest Medical University, Luzhou 646000, China; ^5^Department of Cardiovascular Surgery, The Affiliated Hospital of Southwest Medical University, Luzhou 646000, China; ^6^Cardiovascular and Metabolic Diseases Key Laboratory of Luzhou, Luzhou 646000, China; ^7^Key Laboratory of Medical Electrophysiology, Ministry of Education & Medical Electrophysiological Key Laboratory of Sichuan Province, (Collaborative Innovation Center for Prevention of Cardiovascular Diseases) Institute of Cardiovascular Research, Southwest Medical University, Luzhou 646000, China

## Abstract

**Objectives:**

To investigate the effects of cytotoxin-associated gene A- (CagA-) positive *Helicobacter pylori* on proliferation, invasion, autophagy, and expression of miR-125b-5p in colon cancer cells.

**Methods:**

Colon cancer cells were cocultured with *H. pylori* (CagA+) to analyze the effects of *H. pylori* on miR-125b-5p and autophagy. Colon cancer cells infected with *H. pylori* (CagA+) were mimicked by transfection of CagA plasmid. The effects of CagA on the proliferation, invasion, and autophagy of colon cancer cells were analyzed. Cell counting kit-8 (CCK-8), clone formation, and Transwell assays were used to detect cell viability, proliferation, and invasion ability, respectively. Proteins and miRNAs were detected by western blotting and qPCR, respectively.

**Results:**

*H. pylori* (CagA+) inhibited expression of miR-125b-5p and promoted autophagy in colon cancer cells. MiR-125 b-5p was underexpressed in colon cancer cells after CagA overexpression. CagA promoted colon cancer cell proliferation, invasion, and autophagy. Overexpression of miR-125b-5p inhibited the proliferation, invasion, and autophagy of colon cancer cells and reversed the effects of CagA.

**Conclusion:**

*H. pylori* (CagA+) infection may promote the development and invasion of colon cancer by inhibiting miR-125b-5p.

## 1. Introduction

Colon cancer is a common digestive tract tumor that usually occurs in people aged 40–50 years. According to a report, colon cancer is one of the most common tumors in China, and survey statistics show that the incidence of colon cancer among young people is increasing [1–3]. Despite tremendous breakthroughs in the detection and treatment of colon cancer, the 5-year survival rate of colon cancer patients is still not satisfactory. Indeed, more than 50% of patients with colon cancer have distant metastasis at diagnosis, which is an important factor leading to poor prognosis [4, 5].

*Helicobacter pylori* (*H. pylori*) is the major virulence factor of chronic gastritis and peptic ulcers, which are closely related to the pathogenesis of gastric mucosa lymphoid tissue lymphoma and gastric cancer [6–8]. In recent years, it has been found that *H. pylori* may be associated with the pathogenesis of colon cancer and polyps. Zumkeller et al. first discovered through metastasis analysis that *H. pylori* infection is potentially linked to the pathogenesis of colon cancer and adenomatous polyps [9]. Teimoorian et al. also found that *H. pylori* is associated with colon cancer and adenomatous polyps [10]. The genotype differences of *H. pylori* strains are important factors leading to different clinical outcomes after infection. There is also a higher risk of serious clinical consequences of infection with cytotoxin-associated gene A- (CagA-) positive *H. pylori* than with the negative strain [11–13].

MicroRNAs (miRNAs), small noncoding single-stranded RNAs, consist of approximately 22 nucleotides encoded by an endogenous gene. miRNAs can directly bind to target messenger RNA (mRNA) by recognizing and complementing the 3′-untranslated region (UTR). miRNAs lead to gene degradation or translation, thus downregulating the expression of target genes [14, 15]. Regulation of posttranscriptional gene expression of miRNAs plays important roles in tumorigenesis, metastasis, and drug resistance [16–18]. *H. pylori* may regulate the proliferation of gastric cancer cells by inhibiting miR-152 and miR-200b [19]. The level of miR-490-3p is also associated with the prognosis of patients with gastric cancer caused by *H. pylori* [20]. However, the mechanism of *H. pylori*-induced colon cancer is still unclear.

In this study, it was found that CagA-positive *H. pylori* might promote the proliferation, invasion, and autophagy of colon cancer cells by inhibiting miR-125b-5p, thereby inducing colon cancer.

## 2. Materials and Methods

### 2.1. Cell Culture and Plasmid Transfection

Colon cancer cell lines DLD-1 and SW620 (American Type Culture Collection, USA) were cultured in RPMI-1640 medium containing 10% fetal bovine serum (FBS, Thermo Fisher, Waltham, USA), 50 U/mL penicillin, and 50 *μ*g/mL streptomycin (15070063, Thermo Fisher, Waltham, USA).

Standard *H. pylori* (CagA+, VacA+) NCTCl1637 was provided by the Chinese Center for Disease Control and Prevention. An *H. pylori* suspension with a concentration of 1 × 10^5^ CFU/mL was added at a ratio of 200 : 1 when colon cancer cells were grown to 80% confluence. Cell changes were observed at 24 h after coculture, and the expression of CagA protein was detected by western blotting. The autophagy-related proteins LC3B-II/LC3B-I, Beclin-1, and miR-125b-5p were detected by western blotting and qPCR, respectively.

*H. pylori* (CagA+) infection was simulated by transfection of the CagA plasmid (GenePharma). Transfection was carried out according to the kit instructions. The miR-125b-5p mimic overexpresses miR-125b-5p by plasmid transfection. Colon cancer cell lines DLD-1 and SW620 were divided into four groups: mimic-NC + OE-NC (miR-125b-5p negative control + CagA negative control), mimic-miR-125b-5p + OE-NC (miR-125b-5p overexpression + CagA negative control), mimic-NC + OE-CagA (miR-125b-5p negative control + CagA overexpression), and mimic-miR-125b-5p + OE-CagA (miR-125b-5p overexpression + CagA overexpression). For different experimental groups, 2 *μ*L of Lipofectamine^TM^ 2000 (Invitrogen, Waltham, USA), 40 pmol of CagA plasmid, miR-125b-5p plasmid and negative control (NC) (GenePharma) were mixed in 50 *μ*L of serum-free medium at room temperature for 15 min. The lipid compounds were diluted in 300 *μ*L of serum-free medium and 600 *μ*L of medium containing FBS to produce a 1 mL volume mixture and incubated with the cells at 37°C with 5% CO_2_ for subsequent experiments.

### 2.2. QPCR

Total RNA was obtained using TRIzol (Invitrogen, Waltham, USA). The concentration and purity of the RNA were detected by a NanoDrop2000 spectrophotometer (NanoDrop Technologies, Wilmington, DE, USA). One microgram of RNA was reverse transcribed using a reverse transcription cDNA kit (Thermo Fisher Scientific, Waltham, USA) for the synthesis of cDNA. SYBR Green PCR Master Mix (Roche, Basel, Switzerland) was used to conduct the qPCR experiments using a PCR Detection System (ABI 7500, Life Technology, USA). The PCR cycle was as follows: pretreatment at 95°C for 10 min, followed by 40 cycles of 94°C for 15 s, 60°C for 1 min, 60°C for 1 min, and 4°C for preservation. Comparative cycle threshold (2^−ΔΔ^Ct) analysis was employed to determine the expression of the RNAs [21, 22]. The expression levels of GAPDH and U6 were used for normalization. Primer sequences of the genes used in this work are described in Table 1.

### 2.3. Cell Counting Kit-8 (CCK-8) Assay

The cells were adjusted to a density of 2 × 10^4^ cells/mL and inoculated in 96-well plates (100 *μ*L per well). Forty-eight hours after transfection, 10 *μ*L of CCK-8 (Beyotime Institute of Biotechnology, Beijing, China) was added and cultured at 37°C for 2 h. The optical density (OD) at 450 nm was measured by a microplate reader (Tecan Infinite M200 Microplate Reader; LabX, Männedorf, Switzerland) to calculate the relative cell viability.

### 2.4. Clone Formation Experiment

A total of 1 × 10^3^ cells were inoculated per well into 6-well plates. The cells were cultured in a 5% CO_2_ incubator for 2 weeks at 37°C. After aspirating the medium, 500 *μ*L of methanol solution was added to each well to fix the cells for 15 min, and then 1 mL of crystal violet dye solution was added for 20 min. An automatic image analyzer was used to scan and photograph the cells, and the clone formation numbers were tested.

### 2.5. Transwell Assay

A total of 3 × 10^4^ cells were transferred into the upper chambers of a Transwell apparatus (8 *μ*m, BD Biosciences, CA, USA). The bottom chamber was filled with a complete medium supplemented with 10% FBS. After incubation for 48 h, cells that did not invade through the membrane were swept away. Then, the cells were fixed with 20% methanol and stained with 0.2% crystal violet. Cells invading into the bottom chamber per field were counted under an inverted microscope.

### 2.6. Western Blotting

Protein was extracted by protein lysate (RIPA). A BCA kit was applied to analyze the protein concentration. Protein was separated by SDS-PAGE at 110 V for 100 min and transferred to PVDF membranes. The PVDF membranes were blocked in 5% nonfat milk for 1 h at room temperature. The antibodies (CagA, ab224836, Abcam, San Francisco, USA; Bcl2, ab59348, 26 kD; cyclin D1, ab134175, 34 kD; E-cadherin, ab40772, 97 kD; N-cadherin, ab18203, 130 kD; LC3B-II/LC3B-I, ab48394, 19 kD/17 kD; GAPDH, ab8245,36 kD; Beclin-1, ab207612, 52 kD) were diluted at 1 : 1000 with 5% BSA and added to the cells overnight at 4°C. Then, the secondary antibody (sc-516102/sc-2357; Santa Cruz Biotechnology, Inc. Dallas, TX, USA) was diluted at 1 : 5000 and added to the cells at room temperature for 2 h. Protein blot bands were detected by Pierce™ ECL plus western blotting substrate (Thermo Fisher, Waltham, USA) in ChemiDoc MP (Bio-Rad, California, USA).

### 2.7. Statistical Analysis

All experimental data are presented as the mean ± SD, and *p* < 0.05 was considered statistically significant. All statistical analyses were performed using GraphPad Prism 6.

## 3. Results

*H. pylori* (CagA+) inhibits miR-125b-5p and promotes LC3B-II/LC3B-I and Beclin-1 in colon cancer cells.

**Figure 1:**
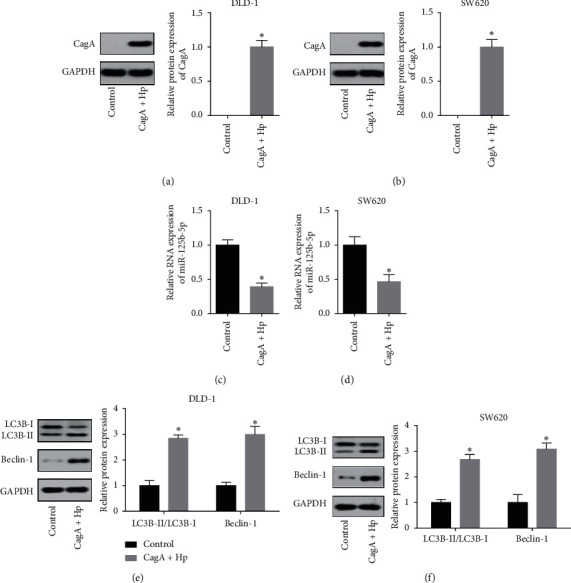
*H. pylori* (CagA+) inhibits miR-125b-5p and promotes LC3B-II/LC3B-I and Beclin-1 in colon cancer cells. (a) and (b) represent CagA protein expression was significantly increased after coculture of both the DLD-1 (a) and SW620 (b) colon cancer cell lines with *H. pylori* (CagA+). (c) and (d) represent that, after coculture with *H. pylori* (CagA+), miR-125b-5p expression was significantly decreased in both DLD-1 (c) and SW620 (d) cells. (e) and (f) represent the expression of the autophagy-related proteins LC3B-II/LC3B-I and Beclin-1 was significantly higher than that in the control group for both DLD-1 (e) and SW620 (f) cells. ^*∗*^*p* < 0.05.

CagA protein expression was significantly increased after coculture of both the DLD-1 (Figure 1(a)) and SW620 (Figure 1(b)) colon cancer cell lines with *H. pylori* (CagA+). After coculture with *H. pylori* (CagA+), miR-125b-5p expression was significantly decreased in both DLD-1 (Figure 1(c)) and SW620 (Figure 1(d)) cells. The expression of the autophagy-related proteins LC3B-II/LC3B-I and Beclin-1 was significantly higher than that in the control group for both DLD-1 (Figure 1(e)) and SW620 (Figure 1(f)) cells. The results indicated that *H. pylori* (CagA+) inhibited the expression of miR-125b-5p and promoted the expression of LC3B-II/LC3B-I and Beclin-1 in colon cancer cells.

CagA overexpression inhibits miR-125b-5p in colon cancer cells.

**Figure 2:**
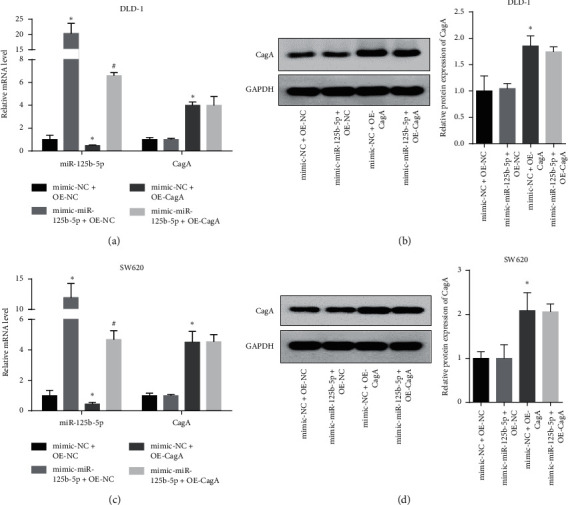
CagA overexpression inhibits miR-125b-5p, while miR-125b-5p does not affect CagA expression in DLD-1 and SW620 colon cancer cells. (a) and (c) represent the transfection of CagA plasmid decreased the miR-125b-5p level, while transfection of miR-125b-5p did not affect the CagA mRNA level in DLD-1 (a) and SW620 (c) cancer cell lines. (b) and (d) represent the transfection of miR-125b-5p did not affect the CagA protein level in DLD-1 (b) and SW620 (d) cancer cell lines. ^*∗*^*p* < 0.05, compared with mimic-NC + OE-NC, and ^#^*p* < 0.05, compared with mimic-NC + OE-CagA group.

An *H. pylori* (CagA+) infection model was constructed by transfecting CagA. The qPCR results showed that transfection of the CagA plasmid increased the expression of CagA and decreased miR-125b-5p, and transfection of miR-125b-5p increased the expression of miR-125b-5p, but it was still lower than that of the CagA negative control and did not affect the expression of CagA in either the DLD-1 or SW620 colon cancer cell lines (Figures 2(a) and 2(c)). The western blot results also showed that overexpression of miR-125b-5p did not affect the expression of the CagA protein in either the DLD-1 or SW620 colon cancer cell lines (Figures 2(b) and 2(d)). This indicated that the transfection experiment was successful. Moreover, overexpression of miR-125b-5p did not affect the infection efficiency of CagA but did reverse the inhibitory effect of CagA on miR-125b-5p.

CagA overexpression promotes the proliferation and invasion of colon cancer cells by inhibiting miR-125b-5p.

**Figure 3:**
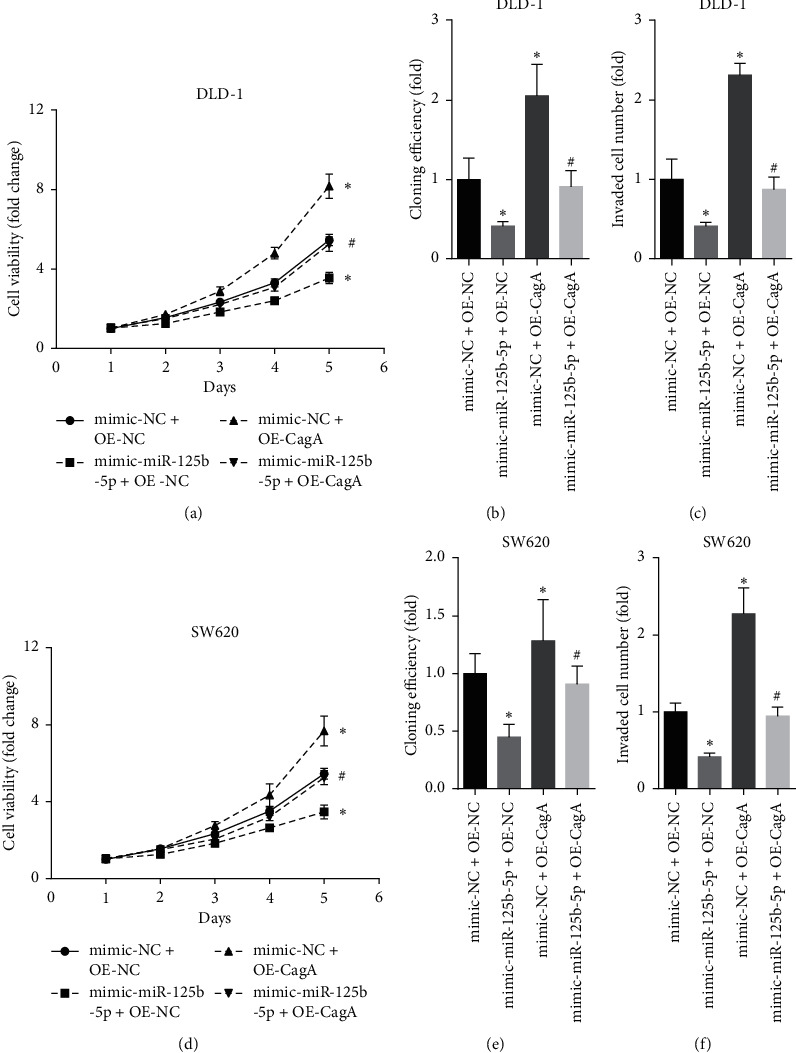
CagA overexpression promotes, while miR-125b-5p inhibits, the proliferation and invasion of colon cancer cells. (a) and (d) represent that on the fifth day, transfection of CagA plasmid significantly increased, while transfection of miR-125b-5p significantly decreased the viability of both DLD-1 (a) and SW620 (d) cells. (b) and (e) represent the transfection of CagA plasmid significantly increased, while transfection of miR-125b-5p significantly decreased the proliferation of both DLD-1 (b) and SW620 (e) cells. (c) and (f) represent the transfection of CagA plasmid significantly increased, while transfection f miR-125b-5p significantly decreased the invasion of DLD-1 (c) and SW620 (f) cells. ^*∗*^*p* < 0.05, compared with mimic-NC + OE-NC, and ^#^*p* < 0.05, compared with mimic-NC + OE-CagA group.

On the fifth day, overexpression of CagA significantly increased the viability of both DLD-1 (Figure 3(a)) and SW620 (Figure 3(d)) cells. miR-125b-5p overexpression significantly decreased the viability of both DLD-1 (Figure 3(a)) and SW620 (Figure 3(d)) cells and reversed the effect of CagA on their viability. Increased levels of CagA also significantly increased the proliferation of both DLD-1 (Figure 3(b)) and SW620 (Figure 3(e)) cells, whereas miR-125b-5p overexpression significantly decreased the proliferation of both DLD-1 (Figure 3(b)) and SW620 (Figure 3(e)) cells and reversed the effect of CagA on their proliferation. CagA overexpression significantly increased the invasion of DLD-1 (Figure 3(c)) and SW620 (Figure 3(f)) cells, and overexpressing miR-125b-5p significantly decreased the invasion of both DLD-1 (Figure 3(c)) and SW620 (Figure 3(f)) cells and reversed the effect of CagA on their invasion.

**Figure 4:**
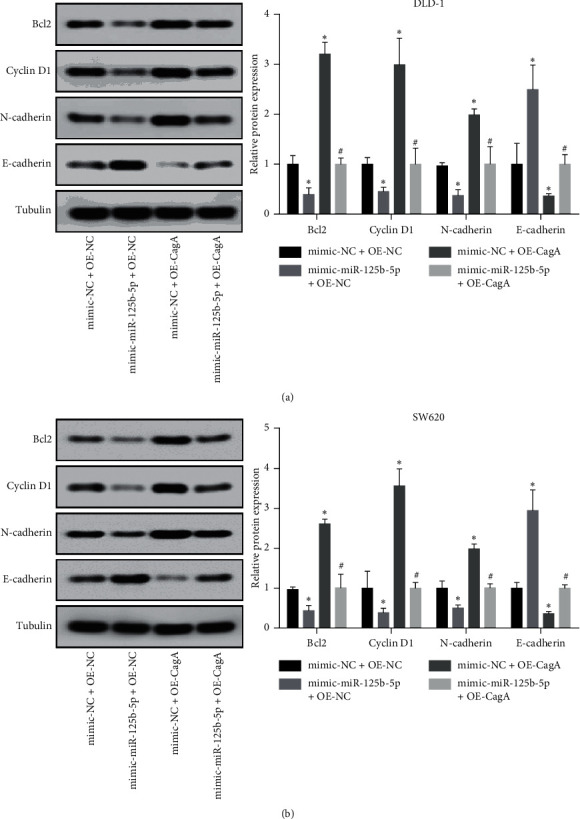
CagA overexpression promotes the proliferation and invasion of colon cancer cells by inhibiting miR-125b-5p. (a) and (b) represent the transfection of CagA plasmid increased the expression of the apoptosis-related protein Bcl2, the proliferation-related protein cyclin D1, and the invasion-related protein N-cadherin but decreased the expression of E-cadherin, while transfection of miR-125b-5p had the opposite effect and reversed the effects of CagA on Bcl2, cyclin D1, N-cadherin, and E-cadherin in both DLD-1 (a) and SW620 (b) cells. ^*∗*^*p* < 0.05, compared with mimic-NC + OE-NC, and ^#^*p* < 0.05, compared with mimic-NC + OE-CagA group.

Higher levels of CagA increased the expression of the apoptosis-related protein Bcl2, the proliferation-related protein cyclin D1, and the invasion-related protein N-cadherin but decreased the expression of E-cadherin in both DLD-1 (Figure 4(a)) and SW620 (Figure 4(b)) cells. Moreover, overexpression of miR-125b-5p had the opposite effect and reversed the effects of CagA on Bcl2, cyclin D1, N-cadherin, and E-cadherin in both DLD-1 (Figure 4(a)) and SW620 (Figure 4(b)) cells. This indicated that CagA overexpression promoted the proliferation and invasion of colon cancer cells by inhibiting miR-125b-5p.

CagA overexpression promotes autophagy in colon cancer cells by inhibiting miR-125b-5p.

**Figure 5:**
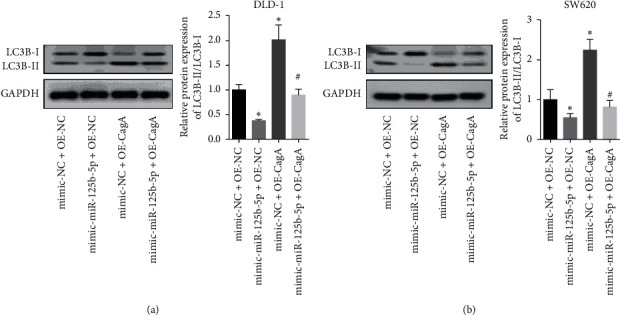
CagA overexpression promotes autophagy in colon cancer cells by inhibiting miR-125b-5p. (a) and (b) represent the transfection of CagA plasmid promoted, while transfection of miR-125b-5p inhibited the expression of LC3B-II/LC3B-I and reversed the effects of CagA on the expression of LC3B-II/LC3B-I in both DLD-1 (a) and SW620 (b) cells. ^*∗*^*p* < 0.05, compared with mimic-NC + OE-NC, and ^#^*p* < 0.05, compared with mimic-NC + OE-CagA group.

Overexpression of CagA promoted the expression of the autophagy-related proteins LC3B-II/LC3B-I in both DLD-1 (Figure 5(a)) and SW620 (Figure 5(b)) colon cancer cells. Moreover, the overexpression of miR-125b-5p inhibited the expression of LC3B-II/LC3B-I and reversed the effects of CagA on the expression of LC3B-II/LC3B-I in both DLD-1 (Figure 5(a)) and SW620 (Figure 5(b)) cells. This further indicated that CagA promoted autophagy by inhibiting the expression of miR-125b-5p, thus promoting the proliferation and invasion of colon cancer cells.

## 4. Discussion

*H. pylori* is considered a class I carcinogen, and its role in gastric cancer has been widely recognized. *H. pylori* also plays a role in other digestive tract tumors [13]. The genotype differences of *H. pylori* strains are important factors leading to different clinical outcomes after infection. The risk of serious clinical consequences with CagA-positive strains is significantly greater than that with CagA-negative strains [23]. Research from Europe and the United States has shown that the CagA gene is present in approximately 50–70% of *H. pylori* strains. The incidence and severity of gastrointestinal ulcers in patients infected with CagA + *H. pylori* are significantly higher than in those infected with CagA strains [24]. Researchers from China also showed that the detection rate of the CagA + strain is as high as 90% in patients with chronic gastritis [25]. After *H. pylori* infection, CagA is injected into the host cell through the CagPAI-type IV secretion system and phosphorylated, causing serious tissue inflammatory damage in the host and leading to abnormal cell function [26]. In addition, studies have confirmed in recent years that *H. pylori* can promote the epithelial–mesenchymal transition [27, 28].

In this study, the effect of CagA + *H. pylori* on colon cancer cells was analyzed. First, it was discovered that *H. pylori* (CagA+) inhibited the expression of miR-125b-5p. Other studies have found that miR-125b-5p plays an important role in the inhibition of breast cancer, gallbladder cancer, esophageal squamous cell carcinoma, and other tumors [29–31]. Second, *H. pylori* (CagA+) infection was induced by transfection of the CagA plasmid, which showed that CagA promoted the expression of proliferation-related proteins and invasion-related proteins, thus promoting the proliferation and invasion of colon cancer cells. Finally, *H. pylori* (CagA+) infection promoted the expression of autophagy-related proteins. However, the overexpression of miR-125b-5p had the opposite effects and reversed the effects of CagA on proliferation, invasion, and autophagy. These results indicated that *H. pylori* (CagA+) might participate in the development and invasion of colon cancer by promoting autophagy, which can be inhibited by miR-125b-5p. Cao's study [32] showed that miR-125b-5p participates in the development of systemic lupus erythematosus and inhibits autophagy by targeting UVRAG. Xiao also reported that miR-125b-5p regulates autophagy [33].

Autophagy is the main pathway through which normal cells resist external stress and stimulation, but it has a dual effect on cancer cells. Autophagy promotes and inhibits the formation and development of tumors and plays different roles in different tumors and different stages of tumor development. In the early stage of tumor growth, the inhibition of autophagy activity can lead to the continuous growth of precancerous cells, indicating the role of autophagy in suppressing tumor growth; in the later stage of tumor growth, the tumor cells in the central ischemic area of the tumor experience poor nutrient status for a long duration. In the hypoxic state, autophagy provides energy support for the growth of tumor cells by degrading macromolecular substances, proteins, and organelles in the cell, which is beneficial to the growth of tumor cells in a hypovascular environment [34]. Additionally, tumor cells can resist inflammatory reactions and acquire drug resistance through autophagy [35].

In conclusion, *H. pylori* (CagA+) inhibits the expression of miR-125b-5p in colon cancer cells and promotes autophagy. Overexpression of miR-125b-5p reverses the role of CagA in promoting the proliferation, invasion, and autophagy of colon cancer cells. This indicates that *H. pylori* (CagA+) infection may promote the development and invasion of colon cancer by inhibiting autophagy, but its specific mechanism needs further study.

## Figures and Tables

**Table 1:** Primer sequences.

Primer name	Sequence (5′-3′)
miR-125b-5p-forward	TCCCTGAGACCCTAACTTGTGA
miR-125b-5p-reverse	AGTCTCAGGGTCCGAGGTATTC
CagA-forward	ATAATGCTAAATAGACAACTTGAGCGA
CagA-reverse	TTAGAATAATCAACAAACATCACGCCAT
U6-forward	CTCGCTTCGGCAGCACA
U6-reverse	AACGCTTCACGAATTTGCGT
GAPDH-forward	GGGAGCCAAAAGGGTCAT
GAPDH-reverse	GAGTCCTTCCACGATACCAA

## Data Availability

All data generated or analyzed during this study are included in this published article.

## References

[1] Chen W. (2015). Cancer statistics: updated cancer burden in China. *Chinese Journal of Cancer Research*.

[2] Chen W., Sun K., Zheng R. (2018). Cancer incidence and mortality in China, 2014. *Chinese Journal of Cancer Research*.

[3] Weinberg B. A., Marshall J. L., Salem M. E. (2017). The growing challenge of young adults with colorectal cancer. *Oncology (Williston Park, N.Y.)*.

[4] Siegel R. L., Miller K. D., Fedewa S. A. (2017). Colorectal cancer statistics, 2017. *CA: A Cancer Journal for Clinicians*.

[5] You Y. N., Rustin R. B., Sullivan J. D. (2015). Oncotype DX colon cancer assay for prediction of recurrence risk in patients with stage II and III colon cancer: a review of the evidence. *Surgical Oncology*.

[6] Kuo S. H., Yeh K. H., Wu M. S. (2017). First-line antibiotic therapy in Helicobacter pylori-negative low-grade gastric mucosa-associated lymphoid tissue lymphoma. *Science Reports*.

[7] Amieva M., Peek R. M. (2016). Pathobiology of helicobacter pylori-induced gastric cancer. *Gastroenterology*.

[8] Espinoza J. L., Matsumoto A., Tanaka H., Matsumura I. (2018). Gastric microbiota: an emerging player in helicobacter pylori-induced gastric malignancies. *Cancer Letters*.

[9] Zumkeller N., Brenner H., Zwahlen M., Rothenbacher D. (2006). Helicobacter pylori infection and colorectal cancer risk: a meta-analysis. *Helicobacter*.

[10] Teimoorian F., Ranaei M., Hajian Tilaki K., Shokri Shirvani J., Vosough Z. (2018). Association of helicobacter pylori infection with colon cancer and adenomatous polyps. *Iranian Journal of Pathology*.

[11] Chen S., Duan G., Zhang R., Fan Q. (2014). Helicobacter pylori cytotoxin-associated gene A protein upregulates *α*-enolase expression via Src/MEK/ERK pathway: implication for progression of gastric cancer. *International Journal of Oncology*.

[12] Ibrahim M., Rafaat T., Abbas A., Masoud H., Salama A. (2014). A novel association between cytotoxin-associated gene A (CagA) positive strain ofHelicobacter pyloriand unexplained recurrent early pregnancy loss. *The European Journal of Contraception & Reproductive Health Care*.

[13] Park J. Y., Forman D., Waskito L. A., Yamaoka Y., Crabtree J. E. (2018). Epidemiology of Helicobacter pylori and CagA-positive infections and global variations in gastric cancer. *Toxins (Basel)*.

[14] Ritchie W. (2017). microRNA target prediction. *Methods in Molecular Biology*.

[15] Mellis D., Caporali A. (2018). MicroRNA-based therapeutics in cardiovascular disease: screening and delivery to the target. *Biochemical Society Transactions*.

[16] Xu J., Zheng J., Wang J., Shao J. (2019). miR-876-5p suppresses breast cancer progression through targeting TFAP2A. *Experimental and Therapeutic Medicine*.

[17] Li R., Teng X., Zhu H., Han T., Liu Q. (2019). MiR-4500 regulates PLXNC1 and inhibits papillary thyroid cancer progression. *Horm Cancer*.

[18] Li M., Gao M., Xie X. (2019). MicroRNA-200c reverses drug resistance of human gastric cancer cells by targeting regulation of the NER-ERCC3/4 pathway. *Oncology Letters*.

[19] Xie G., Li W., Li R. (2017). Helicobacter pylori promote B7-H1 expression by suppressing miR-152 and miR-200b in gastric cancer cells. *PLoS One*.

[20] Qu M., Li L., Zheng W. C. (2017). Reduced miR-490-3p expression is associated with poor prognosis of Helicobacter pylori induced gastric cancer. *European Review for Medical and Pharmacological Sciences*.

[21] Huang M., Kim H. G., Zhong X. (2020). Sestrin 3 protects against diet‐induced nonalcoholic steatohepatitis in mice through suppression of transforming growth factor *β* signal transduction. *Hepatology*.

[22] Zhong X., Huang M., Kim H.-G. (2020). SIRT6 protects against liver fibrosis by deacetylation and suppression of SMAD3 in hepatic stellate cells. *Cellular and Molecular Gastroenterology and Hepatology*.

[23] Hu Y., Zhu Y., Lu N. H. (2017). Novel and effective therapeutic regimens for Helicobacter pylori in an era of increasing antibiotic resistance. *Frontiers in Cellular Infection and Microbiology*.

[24] Nomura A., Stemmermann G. N., Chyou P.-H., Kato I., Perez-Perez G. I., Blaser M. J. (1991). Helicobacter pyloriInfection and Gastric Carcinoma among Japanese Americans in Hawaii. *New England Journal of Medicine*.

[25] Pan Z. J., van der Hulst R. W., Feller M. (1997). Equally high prevalences of infection with cagA-positive Helicobacter pylori in Chinese patients with peptic ulcer disease and those with chronic gastritis-associated dyspepsia. *Journal of Clinical Microbiology*.

[26] Horridge D. N., Begley A. A., Kim J. (2017). Outer inflammatory protein a (OipA) of helicobacter pylori is regulated by host cell contact and mediates CagA translocation and interleukin-8 response only in the presence of a functional cag pathogenicity island type IV secretion system. *Pathog Dis*.

[27] Krzysiek-Maczka G., Targosz A., Szczyrk U. (2018). Role of Helicobacter pylori infection in cancer-associated fibroblast-induced epithelial-mesenchymal transition in vitro. *Helicobacter*.

[28] Li N., Feng Y., Hu Y. (2018). Helicobacter pylori CagA promotes epithelial mesenchymal transition in gastric carcinogenesis via triggering oncogenic YAP pathway. *J Exp Clin Cancer Res*.

[29] Li Y., Wang Y., Fan H., Zhang Z., Li N. (2018). miR-125b-5p inhibits breast cancer cell proliferation, migration and invasion by targeting KIAA1522. *Biochemical and Biophysical Research Communications*.

[30] Yang D., Zhan M., Chen T. (2017). miR-125b-5p enhances chemotherapy sensitivity to cisplatin by down-regulating Bcl2 in gallbladder cancer. *Science Reports*.

[31] Mei L. L., Wang W. J., Qiu Y. T. (2017). miR-125b-5p functions as a tumor suppressor gene partially by regulating HMGA2 in esophageal squamous cell carcinoma. *PLoS One*.

[32] Cao W., Qian G., Luo W. (2018). miR-125b is downregulated in systemic lupus erythematosus patients and inhibits autophagy by targeting UVRAG. *Biomedicine & Pharmacotherapy*.

[33] Xiao C., Wang K., Xu Y. (2018). Transplanted mesenchymal stem cells reduce autophagic flux in infarcted hearts via the exosomal transfer of miR-125b. *Circulation Research*.

[34] Carroll R. G., Martin S. J. (2013). Autophagy in multiple myeloma: what makes you stronger can also kill you. *Cancer Cell*.

[35] Xu L., Zhang X., Li Y. (2016). Neferine induces autophagy of human ovarian cancer cells via p38 MAPK/JNK activation. *Tumor Biology*.

